# Surgical Co-apter and Splints

**Published:** 1848-02

**Authors:** 


15. *Surgical Co-apter and Splints.*—A medical student, Mr. Oliver D. Wilson, of West Boylston, Mass., has invented and secured a patent for reducing dislocations, which he calls the Co-apter, accompanied by a variety of curiously constructed splints, made of iron. He has certainly hit upon something that merits attention. He appears to have been led to the invention in consequence of the high cost of other kinds of surgical adjusters. The new instrument seems to accomplish the same result that is attained by other approved ones, and by a simplicity of mechanism that leads one to wonder that the principle on which it and others operate, was not discovered long ago. Without attempting a description, which we could not easily give, it may be proper to observe that the inventor proposes to publish an account of the peculiar properties of each article, accompanied by drawings, when the subject may again be called up by ourselves.—*Ibid.*

